# SuperNOVA: Semi-Parametric Identification and Estimation of Interaction and Effect Modification in Mixed Exposures using Stochastic Interventions in R

**DOI:** 10.21105/joss.05422

**Published:** 2023-11-05

**Authors:** David McCoy, Alejandro Schuler, Alan Hubbard, Mark van der Laan

**Affiliations:** 1 Department of Biostatistics, University of California Berkeley, Berkeley, CA 94704, U.S.A.

## Abstract

Environmental epidemiology studies aim to understand the impact of mixed exposures on health outcomes while adjusting for covariates. However, traditional statistical methods make simplistic assumptions that may not be applicable to public policy decisions. Researchers are ultimately interested in answering causal questions, such as the impact of reducing toxic chemical exposures on adverse health outcomes like cancer. For example, in the case of PFAS, a class of chemicals measured simultaneously in blood samples, identifying the shifts that result in the greatest reduction in thyroid cancer rates can help more directly inform policy decisions on PFAS. In mixtures, nonlinear and non-additive relationships call for new statistical methods to estimate such modified exposure policies. To address these limitations, the open-source SuperNOVA package has been developed to use data-adaptive machine learning methods for identifying variable sets that have the most explanatory power on an outcome of interest. This package applies non-parametric definitions of interaction and effect modification to these variable sets in a mixed exposure, enabling researchers to explore modified treatment policies using stochastic interventions and answer causal questions. The SuperNOVA software implements the data-adaptive discovery of variable sets and estimation using optimal estimators for stochastic interventions described in our paper “Semi-Parametric Identification and Estimation of Interaction and Effect Modification in Mixed Exposures using Stochastic Interventions” (McCoy et al., 2023).

## Statement of Need

Reliable and accurate estimation of treatment effects is essential in public health and medical research. However, traditional parametric models have limitations, especially when dealing with complex exposure scenarios like mixed exposures or treatments. Semi-parametric statistical methods are necessary to provide unbiased estimates and consistent findings, but they are not always accessible to researchers. The open-source R package SuperNOVA addresses this need by offering a powerful and interpretable framework for estimating non-parametric definitions of interaction and effect modification target parameters. This software reduces the risk of model bias and can help drive faster public health decisions by removing human bias due to model selection. SuperNOVA provides a solution to the limitations of traditional parametric models, enabling researchers to adopt these new methods more easily and achieve more consistent findings in public health and medical research.

## Background

The package SuperNOVA was developed to address the limitations of traditional statistical methods in environmental epidemiology studies. These traditional methods often make overly simplistic assumptions, such as linear and additive relationships, and the resulting statistical quantities may not be directly applicable to public policy decisions. SuperNOVA addresses these limitations by using data-adaptive machine learning methods to identify the variables and variable sets that have the most explanatory power on an outcome of interest. In the variable set discovery, the package builds a discrete Super Learner (Coyle et al., 2020) which is a library of machine learning estimators that uses cross-validation to select the best fitting estimator. This Super Learner is composed of flexible basis function estimators, the best of which is analyzed using ANOVA style analysis to determine the variables that contribute most to the model fit through an F-statistic for basis functions.

The variable sets used in the basis functions drive the target parameters estimated. In the event of basis functions for an individual exposure A, the effects of an individual shift are estimated. For basis function with A and W (a baseline covariate), the effect modification parameter is estimated. Effect modification is defined as an individual shift in a covariate region. If two exposures are included in a basis function $A_{1}$, $A_{2}$ the interaction target parameter is estimated, which is the expected outcome under dual shift of both exposures compared to the sum of expected outcomes given individual shifts independently. For each target parameter we use ensemble machine learning to ascertain the expected outcome under a shift and we use cross-validated targeted maximum likelihood estimation (Hubbard et al., 2016) to debias our initial estimates thereby creating an asymptotically unbiased estimator with minimum variance. When we say shift, we mean a stochastic shift (Díaz & van der Laan, 2012). In this framework we calculate the average outcome after shifting the exposure. A stochastic intervention changes the function that defines the exposure A and its conditional density g(A|W) with a candidate density $g_{A_{\delta }}(A|W)$. The new density defines how the exposure is modified, for example a shift such as in pollution or drug where we increase all exposure by say 100 parts per million and observe the change in outcomes. Stochastic interventions give rise to a counterfactual outcome $Y_{A_{\delta }}:=f_{Y}(A_{\delta },W,U_{Y})$, which is obtained by replacing the natural value of the exposure with a shifted value. The degree of shift δ describes the reduction in exposure, based on the individual’s baseline characteristics W. We can evaluate the causal effect of the intervention by finding the counterfactual mean of the outcome under the modified distribution, $\psi_{0,\delta }=E_{P_{0}}^{A_{\delta }}Y_{A_{\delta }}$. In this way, SuperNOVA allows analysts to explore modified treatment policies and ask causal questions (under assumptions) about the impact of mixed exposures on health outcomes. SuperNOVA uses V-fold cross-validation procedures to avoid over-fitting and incorrect model assumptions. This is done by creating parameter generating samples wherein the variable sets are determined and estimators for nuisance parameters are trained, an estimation sample is then used to estimate the target parameters of interest (Zheng & van der Laan, 2010). Additionally, to avoid positivity violations (user inputs a shift amount that there isn’t enough experimentation in the data to estimate) the shift amount can also be input as a data-adaptive parameter which finds the maximum shift possible for each exposure.

## SuperNOVA’s Scope

The SuperNOVA software package is built for the R language and implements our proposed methodology for estimating modified treatment policies in environmental epidemiology studies for data-adaptively identified variable sets. It is specifically designed to estimate the effects of mixed exposures on health outcomes, while adjusting for covariates and potential confounders.

As input, SuperNOVA takes in variable sets A (exposures), W (covariates), Y (outcome) and a vector of deltas for each exposure in A, representing the degree of shift in each exposure if it is identified as predictive of the outcome. The output of SuperNOVA is a dose-response analysis for variable sets data-adaptively identified in the mixed exposure, estimating the expected outcome under a change in exposure compared to the observed outcome under the observed exposure. Using these shift parameters, users are provided with estimates of non-parametric definitions of interaction and effect modification that are directly informative for public health policy. SuperNOVA is a valuable tool for researchers in many fields who need an interpretable and robust statistical approach to answer modified treatment policy questions, estimates are interpreted as the expected outcome if an exposure was changed by the respective delta.

SuperNOVA is designed to provide analysts with both V-fold specific and pooled results for stochastic intervention causal effects. It integrates with the sl_3_ package (Coyle et al., 2020) to allow for ensemble machine learning to be leveraged in the estimation of nuisance parameters.

## Data Availability

The SuperNOVA package has been made publicly available via GitHub. Use of the SuperNOVA package has been extensively documented in the package’s README and a vignette.
